# Effect of maternal exercises on biophysical fetal and maternal parameters: a transversal study

**DOI:** 10.1590/S1679-45082016AO3758

**Published:** 2016

**Authors:** Caroline Mombaque dos Santos, Wendel Mombaque dos Santos, Francisco Maximiliano Pancich Gallarreta, Camila Pigatto, Luiz Osório Cruz Portela, Edson Nunes de Morais

**Affiliations:** 1 Universidade Federal de Santa Maria, Santa Maria, RS, Brazil.

**Keywords:** Exercise, Pregnancy, Fetal monitoring, Fetus, Exercise test

## Abstract

**Objective:**

To evaluate the acute effects of maternal and fetal hemodynamic responses in pregnant women submitted to fetal Doppler and an aerobic physical exercise test according to the degree of effort during the activity and the impact on the well-being.

**Methods:**

Transversal study with low risk pregnant women, obtained by convenience sample with gestational age between 26 to 34 weeks. The participants carry out a progressive exercise test.

**Results:**

After the exercise session, reduced resistance (p=0.02) and pulsatility indices (p=0.01) were identified in the umbilical artery; however, other Doppler parameters analyzed, in addition to cardiotocography and fetal biophysical profile did not achieve significant change. Maternal parameters obtained linear growth with activity, but it was not possible to establish a standard with the Borg scale, and oxygen saturation remained stable.

**Conclusion:**

A short submaximal exercise had little effect on placental blood flow after exercise in pregnancies without complications, corroborating that healthy fetus maintains homeostasis even in situations that alter maternal hemodynamics.

## RESUMO

**Objetivo:**

Avaliar os efeitos agudos de respostas hemodinâmicas maternas e fetais em gestantes submetidas a Doppler fetal e a um teste de exercício físico aeróbio, de acordo com o grau de esforço durante a atividade e o impacto sobre o bem-estar.

**Métodos:**

Estudo transversal desenvolvido com gestantes de baixo risco, por amostra de conveniência com idade gestacional entre 26 e 34 semanas. As participantes realizam um teste de esforço progressivo.

**Resultados:**

Na artéria umbilical, após sessão de exercício físico, identificou-se a redução do índice de resistência (p=0,02) e do índice de pulsatilidade (p=0,01), mas os demais parâmetros Doppler analisados, além da cardiotocografia e do perfil biofísico fetal, não obtiveram alteração significativa. Os parâmetros maternos obtiveram crescimento linear com a atividade, mas não foi possível estabelecer padrão com a escala de Borg, e a saturação de oxigênio se manteve estável.

**Conclusão:**

O esforço submáximo curto teve pouco efeito sobre o fluxo de sangue da placenta após o exercício em gestações sem complicações, corroborando que o feto hígido mantém a homeostase mesmo em situações que alterem a hemodinâmica materna.

## INTRODUCTION

In Brazil, in 2015, there were approximately 2.7 million pregnancies. There is scarce research about physical exercise during gestation. Even after the 1980’s, when this subject became popular among women and led to more interest in investigating the effects of exercise during pregnancy.^(1,2)^

All over the world, nine countries (Australia, Canada, Denmark, France, Japan, Norway, Spain, United Kingdom, and United States) established guidelines on the subject, and most of them recommend moderate-intensity physical activity during pregnancy, providing advice on how to start an exercise program during this period.^(3)^

Physical exercise is a great ally of public health. The incidence of a sedentary lifestyle during pregnancy ranges from 64.5 to 91.5% and tends to be higher in the third trimester,^(4)^ having a negative impact on live, contributing to the epidemics of obesity and increasing the risk of maternal conditions, such as *diabetes mellitus* and hypertension.^(5,6)^

In the absence of obstetric complications, a total of 30 minutes or more of moderate exercise is recommended on most days of the week.^(7)^ This practice helps reducing low back pain, constipation, and edema; improves disposition, mood, posture; and also reduces insomnia, anxiety and the risk of depression;^(7,8)^ and contributes to increase in muscle mass.^(9)^

Women who maintain an exercise program during pregnancy had no changes in fetal weight and height, and the newborns had better Apgar scores. However, a previous study showed a prevalence of only 11% of pregnant women who complied with the minimum recommended physical activity guidelines.^(10-12)^ We do not have Brazilian data for comparison.

## OBJECTIVE

To evaluate the acute effects of a submaximal exercise test on maternal and fetal hemodynamic responses in low-risk pregnant women.

## METHODS

### Subjects

This is a cross-sectional study, conducted by the Physical Exercise and Pregnancy Group of the *Universidade Federal de Santa Maria* (UFSM), Santa Maria (RS), Brazil. It was reviewed and approved by the Research Ethics Committee of UFSM, acknowledged by the National Committee for Ethics in Research (CONEP-MS), under number CAAE: 07437412.7.0000.5346, opinion number 111.255.

The convenience sample was selected among healthy pregnant women, with a single fetus, who were able to perform physical activity after initial medical examination. They were on prenatal care delivered in Primary Healthcare Units located in the region of the study, during the period of data collection.

The inclusion criteria were early gestational dating (ultrasound imaging in the first trimester); gestational age for inclusion in the protocol, starting at 26 weeks (when it is possible to assess fetal viability); no ultrasonography evidence of fetal malformation, changes in fetal growth, or changes in fetal viability (obstetric and Doppler ultrasound scans performed by the researchers before enrollment in the study); no regular physical activity prior to pregnancy; prenatal care delivered by members of the research team after inclusion in the study.

The exclusion criteria were gestational age outside the range specified in the inclusion criteria; multiple pregnancy; chronic diseases, such as *diabetes mellitus* or hypertension; patients with a history of preterm labor; patients with cervical incompetence or undergoing cerclage, vaginal bleeding or placenta previa; smoking; body mass index >30kg/m^2^ at the beginning of pregnancy; hypertensive disorders of pregnancy and gestational *diabetes mellitus*.

An informed consent form (ICF) was signed by all pregnant women who agreed to participate in the present study.

The volunteers comprised a convenience sample, who underwent aerobic physical exercise sessions at two different moments, with an interval of at least four weeks, within the gestational period proposed for the study.

The maximum gestational age was restricted to 35 weeks for the second data collection, with two collections for each participant. The purpose was to control confounding factors within the sample, and not to compare different moments, since no significant difference was identified on the pilot study.

### Research protocol

Data collection was conducted in the *Laboratório de Fisiologia do Exercício e Performance Humana do Centro de Educação Física e Desportes da UFSM* [Laboratory of Exercise Physiology and Human Performance - Physical Education and Sports Center – UFSM], a safe testing facility that had emergency equipment available, and a team composed of one cardiologist, three gynecologist-obstetricians, and one nurse.

All tests were performed between 8AM and 12AM. The pregnant women were instructed to feed normally, but not to eat or drink anything, except water, one hour before the tests.

The research protocol was conducted in three phases: initial assessment, after resting for 15 minutes, blood pressure (BP) was measured on the left arm with a Premium^®^ manual sphygmomanometer, calibrated by Inmetro, and maternal heart rate (HR) was measured with a RCX5^®^ Polar frequency meter. Both devices were used in all phases of the protocol. Later, with the pregnant woman in a semi-Fowler position, a cardiotocography (CTG) was performed for 20 minutes, using a Bistos BT-300^®^ maternal fetal monitor. The results were classified into categories, ranging from normal, atypical or abnormal, each with different clinical expressions, considering the gestational age upon collection.^(13)^

After that, ultrasound data were obtained at rest to evaluate fetal growth and to detect any growth pattern deviations, which could exclude the pregnant woman from the research. Also collected were data for the fetal biophysical profile (FBP) – that is, fetal movement, respiratory movement, fetal tone, and amniotic fluid index) and Doppler data (Doppler velocimetry of the umbilical artery, middle cerebral artery, the ductus venosus and uterine arteries) to assess fetal viability, using an ultrasound machine GE 4c-RS^®^ Voluson-e with convex transducer. These data were obtained by the same researcher in all collections. Later, the exercise session was conducted (second phase of the protocol). And finally (third phase of the protocol), the same items from the initial assessment were reconsidered immediately after the exercise, except for biometrics.

Throughout the proposed exercise session, the maternal HR was monitored at every minute, and BP and oxygen saturation at every three minutes.

Thus, using a ATL 10200 Inbrasport^®^ treadmill (Super ATL 32km/h model), all patients underwent a progressive treadmill test until volitional fatigue, defined as the voluntary limit beyond which the participant no longer desired to continue, not meaning exhaustion, according to the protocol established by the researchers and based on the Balke et al. modified protocol.^(14)^

The exercise session was divided into six phases, each comprising three minutes: after the initial 3 minutes in the treadmill at 4mph and 0% incline, the speed was increased by 0.5mph and the slope by 3%. The increments were added at every 3 minutes.

Perceived exertion was recorded using the Borg scale^(15)^ for each phase, as well as the mean maternal BP and HR, total time in the treadmill, and distance traveled. Initially, the patients were familiarized with the scale, which ranged from 6 (no exertion) to 20 (maximum exertion) and, for this analysis, it was divided by the researchers into three levels: light to somewhat hard (6-10), hard (11-16), and very hard (17-20).

### Ultrasonography

An obstetric ultrasound examination was performed before each exercise session to ensure normal range of fetal growth, using the growth curve of Hadlock et al.^(16)^

The umbilical artery flow velocity wave was obtained by color Doppler velocimetry in a free loop portion of the cord, and the flow velocity wave of the uterine arteries was obtained in their ascending portion, bilaterally, immediately after their crossing over the iliac artery, before the first ramification. The middle cerebral artery was identified by an axial section of the fetal head, and the flow velocity wave was obtained in the proximal third of the artery using color Doppler. The ductus venosus was obtained at the place where the aliasing was identified, in a median parasagittal section of the fetal abdomen.^(17)^

The evaluation of flow velocity waves on the umbilical, middle cerebral and uterine arteries, as well as the ductus venosus, was accepted when at least four uniform and sequential waves were obtained and stored,^(17)^ with resistance index, pulsatility index, and systole/diastole ratio automatically calculated by the ultrasound device.

### Birth data

Data on birth (gestational age at delivery, type of delivery, birth weight, and Apgar scores) were collected from the Birth Registry Book of the Obstetric Center, *Hospital Universitário de Santa Maria*.

### Statistical analysis

The sample size calculation was performed to obtain a 5% significance, a power of 80%, a 0.45 standard deviation, and 0.35 as the difference to be detected in a two-tailed test,^(18)^ based on previous studies,^(19,20)^ which indicated 26 collections would be sufficient for this study.

To compare the distribution of demographic variables, such as age, number of pregnancies, body mass index, and gestational age at the date of data collection, as well as data referring to birth, test time, and distance traveled, a descriptive analysis of frequency was obtained, with the results expressed in mean or median, minimum value and maximum value.

The normalcy of data was tested by the Shapiro-Wilk test. The paired *t* test was used to compare all variables obtained during the protocol, before and after physical exercise.

The descriptive exploratory test, with the variables mean maternal BP and HR as the dependent list, and the Borg scale as the factor list, in addition to velocity and incline increments, was used to assess in which parameters the exercise could be considered hemodynamically safe, and if it was associated with the Borg scale.^(15)^

The statistical significance was set at p<0.05 and statistical analyses were performed using the Statistical Package for the Social Sciences (SPSS) version 21.0.

## RESULTS

The participants (n=28) had a mean age of 26±6.9 years and mean gestational age upon data collection of 30.51±3.3 weeks. All Cesarean sections were performed due to obstetric indication. The other baseline characteristics of the population are shown on tables 1 and 2.

**Table 1:** Baseline characteristics of pregnant women undergoing submaximal exercise test

Maternal characteristics	(n=28)
Age in full years, mean (SD)	26 (±6.9)
Number of pregnancies, median (1st and 3rd quartiles)	1 (1-4)
Body mass index in kg/m^2^, mean (SD)	23.7 (±3.2)
Gestational age in weeks upon collection, mean (SD)	30.5 (±3.3)
Vaginal delivery (%)	42.9

SD: standard deviation.

**Table 2:** Baseline characteristics of newborns of pregnant women undergoing submaximal exercise test

Newborn characteristics	
Weight (g), mean (SD)	3.028 (±459.1)
Gestational age upon delivery, in weeks, mean (SD)	39 (±0.9)
Apgar score at 1 minute, median (minimum-maximum)	9 (7-10)
Apgar score at 5 minutes, median (minimum-maximum)	10 (9-10)

SD: standard deviation.

All CTG performed before and after the study protocol were classified as category 1.

No significant difference (p=0.28) was observed when fetal HR was assessed before (mean of 142.0±8.4 beats/minute) and after the exercise test (mean of 144.0±9.2 beats/minute). There was a significant difference (p=0.002) in the mean maternal HR and BP before and after the exercise test, showing an increase in both: initial mean maternal HR of 82.5±9.6bpm and final mean maternal HR of 144.8±33.1 bpm; and initial mean blood pressure of 81.4±9.6mmHg and final mean blood pressure of 93.8±20.1mmHg.

No deviations were detected in the fetal growth pattern of the pregnant women enrolled in the research.

The parameters evaluated in the FBP did not present changes with the exercise test. On the other hand, vasodilatation was demonstrated in the umbilical artery after a physical exercise session, due to reduction in the resistance index (p=0.02) and in the pulsatility index (p=0.01). Nonetheless, no changes were observed in the mean pulsatility index of the uterine arteries and in the pulsatility index of the ductus venosus, when comparing the values before and after the exercise test (Table 3).

**Table 3:** Doppler flowmetry parameters before and after submaximal exercise test in pregnant women

Doppler flow variable	Exercise test		p value*

	Before	After	
Pulsatility index of the right uterine artery	0.76	0.74	0.65
Pulsatility index of the left uterine artery	0.80	0.76	0.43
Resistance index of the umbilical artery	0.65	0.62	0.02**
Pulsatility index of the umbilical artery	1.06	0.96	0.01**
Umbilical artery systole/diastole	3.00	2.78	0.06
Resistance index of the middle cerebral artery	0.85	0.85	0.88
Pulsatility index of the middle cerebral artery	2.07	2.09	0.82
Middle cerebral artery systole/diastole	8.23	8.58	0.64
Pulsatility index of the ductus venosus	0.34	0.28	0.10

* Paired *t* test; ** significant p value.

The oxygen saturation maintained values of 99 to 100% in all pregnant women, independent of the protocol phase and of the degree of effort perceived by the participants. The mean maternal BP and HR increased progressively with the advancement of the protocol steps, but did not present a linear relation with the Borg scale levels reported by the study subjects. The mean effort time of the protocol was 11.41±4.23 minutes, with a mean distance of 0.9±0.42km.

## DISCUSSION

A progressive increase in maternal HR and mean BP was observed according to the intensity of the test, but the oxygen saturation remained stable. This is due to the fact that systemic adaptations to exercise differ quantitatively when performed by trained or sedentary organisms, and this deserves attention during pregnancy.^(21,22)^ The concern of professionals is exactly when appropriate compensatory mechanisms that ensure homeostasis do not occur, demonstrating the importance of monitoring physical exercises during pregnancy.

The fetal heart rate did not present statistical difference before and after the exercise test proposed. The fetal physiological response to maternal stimulation, or to release of vasoactive hormones not metabolized by the placenta, often shows transient fetal tachycardia after maternal effort.^(23,24)^ It is noteworthy that the fetal HR returns to baseline levels five minutes after the end of a moderate intensity aerobic activity.^(25,26)^ Therefore, the findings of this research are probably due to the fact that the variable was re-evaluated after a period longer than this, respecting the method of study.

To complement this result, control CTGs were performed before and after the activity. This exam is designed to assess fetal well-being and consists of a continuous and simultaneous recording of fetal heart rate, fetal movements, and uterine contractions. The consensus about CTG is that the presence of acceleration in response to somatic movement suggests adequate intrauterine oxygenation, and rules out fetal hypoxia.^(24)^ All CTG performed in the study were in category 1,^(13)^ *i.e.*, the fetus was found to be reactive (≥2 transient accelerations/20 minutes), and this confirms that a healthy fetus is able to maintain its hemodynamic conditions despite maternal effort.

There was no difference before and after the exercise test on a treadmill in BFP, which is a method that assesses fetal movements, fetal tone, respiratory movements and amniotic fluid volume, and is used for the same purpose as the CTG, *i.e.*, to assess the risk of fetal hypoxia.^(24)^

In addition to CTG and PBF, Doppler velocimetry has gained ground in the evaluation of fetal viability, and the resistance and pulsatility indexes and the systole/diastole ratio of the vascular segments were studied to complement the fetal evaluation after the exercise test.

Similar studies have concluded that, during normal pregnancy, a short submaximal exercise session has little effect on placental blood flow after exercise,^(23,27,28)^ as it was found in this study, in which the uterine arteries showed no difference, and the umbilical artery revealed vasodilation with no clinical consequences, probably due to a fetal mechanism to ensure homeostasis.^(24)^

Although we have identified a fetal cerebral vasodilation in response to hemoglobin desaturation during an activity,^(23)^ the middle cerebral artery Doppler velocimetry indexes showed no significant change after the exercise test, in our study. Note should be made to the difference of methods used in each study, for this may affect the outcomes and make comparisons difficult.

The ductus venosus is essential for supplying oxygenated blood directly to the fetal heart, and its relation with maternal exercise is still incipient. However, in complication-free pregnancies, there is no impact on its hemodynamics,^(29)^ as it was found in this experiment.

Maternal exercise should be appropriate to the individual needs of each pregnant woman, and walking on a treadmill is a comfortable rhythmic activity, in which it is easy to quantify time and quality,^(25)^ as evidenced by this study.

In this study, the use of the Borg scale as an aid for monitoring aerobic activities, which should reach between 12 and 14 points, as pointed out by other studies,^(25)^ did not show an increasing relation between the maternal hemodynamic parameters and its stages, probably due to the previous physical conditioning of the pregnant women. In a sedentary population, heart rate monitoring does not always show a significant relation with exercise intensity.^(26)^

However, the use of perceived exertion, which guarantees the subjective identification of a strenuous exercise, such as when the pregnant woman can no longer speak while performing it, although it does not follow a linear pattern with the maternal heart rate, for example, is justified during an exercise in which maternal monitoring is not possible by other methods.^(26)^

The practice of physical exercise during pregnancy is part of the health promotion scenario and acts as a coadjuvant in the treatment of diseases. In the present study a treadmill stress test was not able to produce acute negative effects on fetal hemodynamics.^(25)^

Nonetheless, some limitations should be described. The favorable fetal outcomes cannot be attributed to the type of research; follow-up programs are necessary to seek results in populations with different characteristics from those studied, and to decrease the mystique of fear in prescribing exercise during pregnancy.

## CONCLUSION

Low-risk pregnant women undergoing submaximal exercise test presented minimal hemodynamic changes, without affecting fetal parameters.
